# The Role of Surgery in Patients with COVID-19-Related Thoracic Complications

**DOI:** 10.3389/fsurg.2022.867252

**Published:** 2022-05-24

**Authors:** Federico Raveglia, Marco Scarci, Arianna Rimessi, Riccardo Orlandi, Paola Rebora, Ugo Cioffi, Angelo Guttadauro, Enrico Ruffini, Mauro Benvenuti, Giuseppe Cardillo, Davide Patrini, Fernando Vannucci, Nasser Yusuf, Pramoj Jindal, Robert Cerfolio

**Affiliations:** ^1^ Thoracic Surgery, San Gerardo Hospital, ASST-Monza, Monza, Italy; ^2^ Bicocca Bioinformatics, Biostatistics and Bioimaging Centre – B4, School of Medicine and Surgery, University of Milano-Bicocca, Monza, Italy; ^3^ University of Milan, Milan, Italy; ^4^ Istituti Clinici Zucchi, University of Milano-Bicocca, Monza, Italy; ^5^ Thoracic Surgery, San Giovanni Battista Molinette Hospital, Turin, Italy; ^6^ Thoracic Surgery, ASST Spedali Civili, Brescia, Italy; ^7^ Thoracic Surgery, San Camillo Forlanini Hospital, Rome, Italy; ^8^ Thoracic Surgery, University College London Hospitals, London, United Kingdom; ^9^ Thoracic Surgery, Hospital Federal do Andaraí, Rio de Janeiro, Brasil; ^10^ Thoracic Surgery, Chest Hospital, Calicut, India/Sunrise Hospital, Kochi, India; ^11^ Thoracic Surgery, Sir Ganga Ram Hospital, New Delhi, India; ^12^ Thoracic Surgery, NYU Langone Health, New York, NY, United States

**Keywords:** COVID-19, complications, thoracic surgery, mortality, surgery

## Abstract

**Objective:**

Patients with several thoracic complications induced by SARS-CoV-2 infection may benefit from surgery, but its role in this condition is largely unknown, and many surgeons’ advice against any surgical referrals. Our aim is to investigate the efficacy and safety of surgery in COVID-19 patients with thoracic complications requiring surgery.

**Methods:**

We designed a multicenter observational study, involving nine thoracic surgery departments, evaluating patients who developed thoracic complications in hospital, surgically managed from March 1, 2020, to May 31, 2021. An overall 30-day mortality was obtained by using the Kaplan–Meier method. Multivariable Cox regression model and logistic models were applied to identify the variables associated with mortality and postoperative complications.

**Results:**

Among 83 patients, 33 (40%) underwent surgery for complicated pneumothorax, 17 (20.5%) for pleural empyema, 13 (15.5%) for hemothorax, 8 (9.5%) for hemoptysis, 5 patients (6%) for lung abscess, 4 (5%) for infected pneumatoceles, and 3 (3.5%) for other causes. Within 30 days of surgery, 60 patients (72%) survived. At multivariable analysis, age (HR 1.05 [95% CI, 1.01, 1.09], *p* = 0.022), pulmonary hypertension (HR 3.98 [95% CI, 1.09, 14.5], *p* = 0.036), renal failure (HR 2.91 [95% CI, 1.19, 7.10], *p*-value 0.019), thoracotomy (HR 4.90 [95% CI, 1.84, 13.1], *p*-value 0.001) and infective affections (HR 0.17 [95% CI, 0.05, 0.58], *p*-value 0.004) were found to be independent prognostic risk factors for 30-day mortality. Age (OR 1.05 [95% CI, 1.01, 1.10], *p* = 0.023) and thoracotomy (OR 3.85 [95% CI, 1.35, 12.0] *p* = 0.014) became significant predictors for 30-day morbidity.

**Conclusion:**

Surgical management of COVID-19-related thoracic complications is affected by high mortality and morbidity rates, but a 72% survival rate still seems to be satisfactory with a rescue intent. Younger patients without pulmonary hypertension, without renal insufficiency and undergoing surgery for infectious complications appear to have a better prognosis.

## Introduction

In late 2019, the world was overwhelmed by the rise of a new respiratory infection called SARS-CoV-2, which has since rapidly spread around the world. By November 2021, there had been more than 252,000,000 confirmed cases and over 5,000,000 deaths worldwide (1, 2).

Significant efforts have been made to better understand this infection’s natural history and its optimal management. Mild COVID-19 disease condition can cause symptoms such as fever, cough, and shortness of breath as well as fatigue and muscle aches. However, severe and critical illnesses are characterized by dramatic and life-threatening symptoms (3). Among these, the most common is pneumonia, which rapidly progresses to ARDS (acute respiratory distress syndrome) (4); but pneumatoceles, parenchymal air-filled cysts, pneumothorax and hemotysis seem to occur more likely in COVID-19 patients as well (5).

The second common critical condition is blood hypercoagulability, probably caused by vascular endothelial cell injury, which manifests with a higher incidence of venous and arterial thrombosis and pulmonary embolism requiring prolonged anticoagulant prophylactic treatment; these conditions cause more likely adverse events such as unexpected hematoma and intrapleural bleeding.

Lastly, these patients often develop co-infections, which lead to further complications such as empyema and septic shock.

The above conditions often require a surgical approach, but based on published data reporting high early postoperative morbidity and mortality (6), this is often a huge challenge.

Therefore, the question is whether salvage surgery should be always excluded or should be considered when it represents the only remaining effective option. In the absence of solid data and recommendations, this is a challenging question for thoracic surgeons.

We designed a multicenter study to record the experiences of several worldwide high-volume thoracic departments. Our objective was to investigate the efficacy and safety of surgery in COVID-19 patients who developed thoracic complications requiring operative management. Our first endpoint was to estimate postoperative survival at 30 days; the secondary endpoints were incidence of postoperative complications and prognostic factors for 30 days’ mortality and morbidity.

## Materials and Methods

### Study Design

We have designed an observational retrospective multicenter international study, involving nine experienced thoracic surgery departments, in five different countries:

- San Gerardo Hospital, Monza, Italy, (coordinator center);
- San Giovanni Battista Molinette Hospital, Turin, Italy;
- Spedali Civili Hospital, Brescia, Italy;
- San Camillo Forlanini Hospital, Rome, Italy;
- University College London Hospitals, London, United Kingdom;
- NYU Langone Health, New York, USA;
- Hospital Federal do Andaraí, Rio de Janeiro, Brazil;
- Chest Hospital, Calicut, India;
- Sir Ganga Ram Hospital, New Delhi, India.

The study was approved by the research ethics committee at the lead center (San Gerardo Hospital, Monza; registration number 3822, October 19, 2021) and was conducted in accordance with the Declaration of Helsinki. At the other centers, local principal investigators were tasked with getting approval. Given the emergency situation leading to surgical treatment and the need to collect data, the IRB decided that consent could be waived.

### Patient Population

The study population consisted of patients who developed in-hospital COVID-19 thoracic complications. The inclusion criteria were as follows: (1) age >18 years old; (2) molecular diagnosis of SARS-CoV-2 infection through nose-pharyngeal swab or bronchoalveolar lavage via real-time PCR analysis; (3) hospital admission because of clinical/radiological diagnosis of pneumonia; (4) onset of thoracic complications during hospitalization or prolonged hospitalization requiring thoracic surgical procedures (we included both life-threatening emergencies and situations in which medical treatment was ineffective); (5) hospital admission from March 1, 2020 to May 31, 2021.

### Surgical Procedures

Thoracic complication is defined as any condition involving the thorax, which is a direct or indirect consequence of COVID-19, including either pathology strictly related to the infection or the iatrogenic effects of therapeutic attempts to treat it. Because of the wide span of diagnosis, the novelty of this pathology and the different protocols adopted by participating centers, it was not possible to identify common criteria for surgical indications. A wide variety of surgical procedures were included. Patients who had undergone chest tube placement alone were not included in the study.

### Data Collection

Data were retrospectively collected in a dedicated password-protected database. Demographic, anamnestic, clinical, surgical, and outcome-related variables were derived from medical and nursing records, laboratory reports, and radiological findings. Patients were followed up to discharge or to 30 days after surgery if discharge occurred before (7).

Follow-up consisted of an outpatient clinic visit, including physical examination and chest x-ray; patients discharged with long-COVID, who were under quarantine at home, according to the regulations in force, were tele medically evaluated.

### Outcomes

The primary endpoint was postoperative survival at 30 days from surgery. The secondary endpoints were postoperative complications. The postoperative complications were graded according to the Thoracic Morbidity and Mortality Classification System from grade I (not needing treatment or intervention) to grade V (leading to death).

### Statistical Analysis

The baseline characteristics of the patients were described in terms of median (I and III quartiles) and frequencies (percentage). Cumulative incidence of in-hospital mortality and discharge was estimated by using the Aalen–Johansen estimator. Overall mortality at 30 days was obtained by the Kaplan–Meier method and compared among groups by using the log-rank test. In order to account for time to death, a multivariable Cox regression model was applied to identify the variables associated with mortality 30 days after surgery. A multivariable logistic model was applied to identify the variables associated with postoperative complications—these included clinical parameters (age, sex, and maximum ventilator support)—and the variables associated with outcomes at the unadjusted analyses (renal insufficiency, pulmonary hypertension, and infective affection (e.g., empyema/pneumatoceles) compared with the other ones (e.g., pneumothorax, hemothorax, etc.) and the type of surgery). The results were presented in terms of hazard ratios (HRs) and odds ratios (ORs) with 95% confidence intervals (CI). Data were analyzed using R software (Version 4.0.3).

## Results

### Population

A total of 83 patients were enrolled in the study, drawn from intensive care, pulmonology and internal medicine departments at different levels of care. All of them were still positive for SARS-Cov-2 RT-PCR at the time of surgery. Anamnestic data are reported in Table 1.

**Table 1:** Anamnestic data of the whole population from 9 different worldwide thoracic surgery departments.

Variable	*N* (%)
Age (years, median [I–III quartiles])	64 [53–70]
Males	62 (75)
Smoking history^a^	
Current	20 (29)
Former	21 (30)
Never	29 (41)
COPD/asthma	26 (31)
Diabetes mellitus	28 (34)
Renal insufficiency	16 (19)
Cardiovascular	44 (53)
Arrhythmia	5 (6)
Hypertension	23 (28)
Hypertension, hyperlipidemia	1 (1)
Hypertension, stroke	1 (1)
Myocardial infarction	6 (7)
Peripheral artery disease	3 (4)
Stroke	2 (2)
Other	3 (4)
Pulmonary hypertension	7 (8)
Preoperative ventilation	
Endotracheal intubation	42 (51)
NIV CPAP	4 (5)
Venturi mask/HFNC	4 (5)
Nasal cannula	11 (13)
Room air	11 (13)
Preoperative chest tube placement	60 (72)
Pulmonary embolism	19 (23)

*COPD, Chronic Obstructive Pulmonary Disease*.

^a^ *Missing in 13 patients*.

### Surgery

The median time that elapsed between COVID-19 diagnosis and surgery was 21 days, with 85% of subjects with time lower than 58 days. All of the patients reporting with empyema was a consequence of pneumonia-related complicated pleural effusion.

Indications for surgery, surgical procedures and their combinations are summarized in Table 2. The median *post-operative* hospital stay was 12 days. A total of 91 surgical procedures was performed on the 83 patients. A total of eight patients (10%) underwent resurgery, all by thoracotomy. One patient was treated for intractable atelectasis (12.5%), managed through pneumonectomy, 1 had empyema (12.5%), treated through decortication and lung resection, 1 had bronchopleural fistula (12.5%), handled through stump repair, 3 patients had hemothorax (37.5%), managed through toilette and hemostasis, 1 had persistent air leak, treated through wedge resection, and 1 had pulmonary infarction, handled through lobectomy.

**Table 2:** Indications for surgery, surgical procedures performed and surgical techniques adopted, in details.

Indication for surgery	Surgical procedures	Number of cases	Surgical technique	
			VATS/RATS	OPEN
Pneumothorax (*n* = 33)	Pleurodesis	18	18	0
	Pleurodesis + wedge resection	9	9	0
	Pleurodesis + decortication	1	1	0
	Wedge resection	2	1	1
	Wedge resection + decortication	3	1	2
Empyema (*n* = 17)	Decortication + pleural toilette	12	6	6
	Decortication + Wedge resection	4	4	0
	Pleural toilette	1	1	0
Hemothorax (*n* = 13)	Pleural toilette	5	1	4
	Pleural toilette + decortication	5	4	1
	Pleural toilette + wedge resection	1	0	1
	Decortication + wedge resection	2	2	0
Hemoptysis (*n* = 8)	Pneumonectomy	3	0	3
	Lobectomy	4	0	4
	Bilobectomy	1	0	1
Lung abscess (*n* = 5)	Lobectomy	3	1	2
	Wedge resection	1	0	1
	Bilobectomy	1	0	1
Pneumatocele (*n* = 4)	Decortication + Pleural toilette	1	0	1
	Decortication + wedge resection	1	0	1
	Wedge	2	1	1
Pulmonary infarction (*n* = 1)	Wedge resection + pleural toilette	1	0	1
Hematoma (*n* = 1)	Pleural toilette	1	0	1
Pleural effusion (*n* = 1)	Pleural toilette + pleurodesis	1	1	0

### Survival

**Figure 1:**
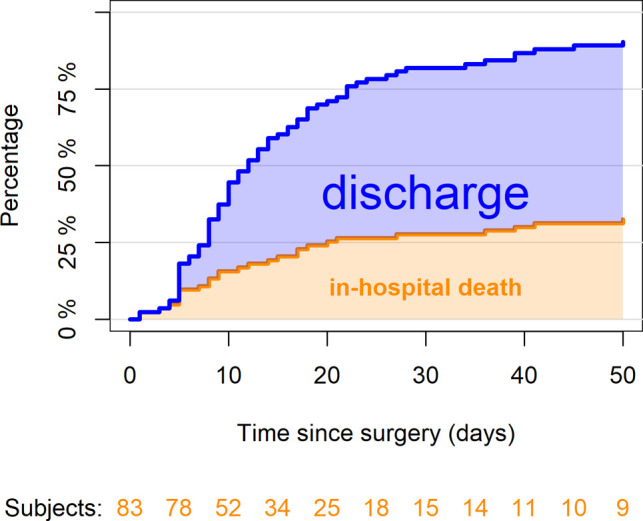
Percentages of patients who died in hospital or who were discharged by the time since surgery (*n* = 83). At 30 days since surgery, 23 patients (28%, 95% CI, 18–37%) died, 45 (54%, 95% CI, 44–65%) were discharged and the remaining 15 were still recovering in hospital. The confidence limits are explained in a supplementary table (Table 7).

Figure 1 reports in-hospital mortality and discharge among the 83 enrolled patients. At 30 days from surgery, 23 patients (28%, 95% CI, 18–37%) died, 45 (54%, 95% CI, 44–65%) survived and were discharged and the remaining 15 were still recovering in hospital; 5 patients died after 30 days during hospitalization; no discharged patient died at home within 30 days. Among the 23 patients who died within 30 days, 13 (16%) died because of postoperative complications, whereas 10 (12%) died for reasons apparently unrelated to surgery and rather linked to the underlying COVID-19 or the disease already present before intervention (Table 3).

**Table 3:** Causes of death—anytime (*n* = 28 death).

Causes of death	Postoperative complications (Clavien V)	Other than postoperative complications	Total
	*n* (%)	*n* (%)	*n* (%)
ARDS	2 (14)	4 (29)	6 (21)
Chest infection	0 (0)	1 + **1** = 2 (14)	2 (7)
Hemorragic shock	0 (0)	**1** (7)	1 (4)
MOF	7 (50)	3 + **1** = 4 (29)	11 (39)
Sepsis	4 + **1** = 5 (36)	2 + **1** = 3 (21)	8 (29)
	13 + **1** = 14 (100)	10 + **4** = 14 (100)	28 (100)

*Among the 23 patients who died within 30 days, 13 died of postoperative complications, whereas 10 died of reasons apparently linked to underlying COVID-19 or the disease already present before intervention.*

a *In bold: deaths occurred after day 30.*

Single pathologic afflictions outcomes are reported in Table 4.

**Table 4:** Outcomes, in terms of complications and the number of deaths, for every single pathologic affection investigated.

	Hospital outcome			
Pathologic affection	Total number of subjects	Number of complications	Number of deaths	Number of patients discharged
Pneumothorax	33	10	12	21
Empyema	17	9	4	13
Hemothorax	13	5	7	6
Hemoptysis	8	4	3	5
Lung abscess	5	3	2	3
Pneumatoceles	4	1	0	4
Hemathoma	1	0	0	1
Pulmonary infarction	1	1	0	1
Pleural effusion	1	0	0	1

At unadjusted analysis, age over 70 (53% vs. 20%, *p*-value 0.017), anamnestic renal insufficiency (69% vs. 18%, *p*-value <0.0001) and anamnestic pulmonary hypertension (53% vs. 25%, *p*-value = 0.031) were positively associated with 30 days’ mortality. Moreover, patients undergoing thoracotomy had worse survival rates than those operated through a minimally invasive approach (47% vs. 16%, *p*-value 0.007). Finally, the survival rates were lower in patients who experienced postoperative complications than in those who did not (50% vs. 12%, *p* < 0.001). On the other hand, gender, days until surgery, smoking history, diabetes, ventilation before surgery, surgical indication and surgical procedures were all not statistically associated with 30-day mortality. All investigated variables are described in Table 5A.

**Table 5A:** Unadjusted univariable analysis of 30-day mortality factors.

Variables	Strata	*n*.	Number of deaths	30-day mortality % (95% CI)	*p*-Value
Gender	F	21	7	33 (10–51)	0.82767
	M	62	16	26 (14–36)	
Age	≤70	64	13	20 (10–30)	0.01677
	>70	19	10	53 (24–71)	
Days since surgery	≤30	51	16	31 (17–43)	0.654265
	>30	32	7	22 (6–35)	
Smoke	Current	20	6	30 (7–47)	0.939265
	Former	21	5	24 (3–40)	
	Non-smoker	29	9	31 (12–46)	
COPD/asthma	No	57	15	26 (14–37)	0.924128
	Yes	26	8	31 (11–46)	
Diabetes mellitus	No	55	14	25 (13–36)	0.817437
	Yes	28	9	32 (12–47)	
Renal insufficiency	No	67	12	18 (8–27)	<0.0001
	Yes	16	11	69 (35–85)	
Cardiovascular	No	39	8	21 (7–32)	0.347753
	Yes	44	15	34 (18–47)	
Pulmonary hypertension	No	76	19	25 (15–34)	0.031513
	Yes	7	4	57 (0–82)	
LMWH	None	13	5	38 (5–60)	0.727392
	Prophylaxis	47	11	23 (10–35)	
	Therapeutic Anticoagulation	23	7	30 (9–47)	
Ventilation before surgery	Room air + venturi + lowflow	26	3	12 (0–23)	0.144558
	cPAP	15	4	27 (0–46)	
	Endotracheal intubation	42	16	38 (22–51)	
Chest tube	No	23	4	17 (0–32)	0.439143
	Yes	60	19	32 (19–42)	
CT scan	No	2	0	0	0.716531
	Yes	81	23	28 (18–38)	
Pulmonary embolism/DVT	No	64	17	27 (15–37)	0.865269
	Yes	19	6	32 (7–50)	
Diagnosis for Surgery	Empyema	17	2	12 (0–26)	0.493004
	Hematoma	1	0	0	
	Hemoptysis	8	3	38 (0–63)	
	Hemothorax	13	6	46 (11–67)	
	Other	6	2	33 (0–62)	
	Pneumatocele	4	0	0	
	Pneumothorax	33	10	30 (13–44)	
	Pulmonary infarction	1	0	0	
Diagnosis for surgery	Empyema/pneumatocele	21	2	10 (0–21)	0.109784
	Others	62	21	34 (21–45)	
Decortication	N	56	16	29 (16–39)	0.984531
	Y	27	7	26 (7–41)	
Lobectomy	N	74	20	27 (16–36)	0.947209
	Y	9	3	33 (0–58)	
Pleuralbiopsy	N	77	20	26 (16–35)	0.408963
	Y	6	3	50 (0–78)	
Pleurodesis	N	54	18	33 (19–45)	0.289225
	Y	29	5	17 (2–30)	
Pneumonectomy	N	80	21	26 (16–35)	0.32245
	Y	3	2	67 (0–93)	
Toilette	N	60	17	28 (16–39)	0.988777
	Y	23	6	26 (6–42)	
Wedge resection	N	61	17	28 (16–38)	0.986537
	Y	22	6	27 (6–44)	
Surgical technique	RATS/VATS	51	8	16 (5–25)	0.006655
	Thoracotomy	32	15	47 (26–62)	
Complications	No	49	6	12 (3–21)	0.000795
	Yes	34	17	50 (30–64)	

*Abbreviations: LMWH, Low-Molecular-Weight Heparin; DVT, Deep Vein Thrombosis.*

At multivariable analysis (Table 5B), age, renal insufficiency, pulmonary hypertension and the thoracotomic approach were found to be independent prognostic risk factors for 30-day mortality. In particular, the older the patient, the higher was the risk of mortality (HR 1.05 [95% CI, 1.01, 1.09], *p*-value 0.022); renal insufficiency was confirmed as a statistically significant prognostic factor in adjusted analysis. In our series, patients who already suffered kidney failure at the time of surgery and those with impaired renal function presented a higher mortality (HR 2.91 [95% CI, 1.19, 7.10], *p*-value 0.019). Lastly, pulmonary hypertension (HR 3.98 [95% CI, 1.09, 14.5], *p*-value 0.036) and thoracotomy (HR 4.90 [95% CI, 1.84, 13.1], *p*-value 0.001) were also associated with a lower 30-day survival. Preoperative endotracheal intubation failed to reach statistical significance, although showing a clear trend toward a higher 30-day mortality (HR 2.85 [95% CI, 0.74, 11], *p*-value 0.13). Finally, with regard to the different affections surgically treated, infective pathologies (e.g., empyema/pneumatoceles/lung abscess) compared with others (e.g., pneumothorax, hemothorax, etc.) resulted in a significant protective factor, ensuring a lower 30-day mortality (HR 0.17 [95% CI, 0.05, 0.58], *p*-value 0.004).

**Table 5B:** Multivariable Cox regression on 30-day mortality (*n* = 83, 23 deaths).

Variable	HR (95% CI)	*p*-Value
Age (per year)	1.05 (1.01–1.09)	0.022
Gender (males vs. females)	1.29 (0.47–3.50)	0.6
Renal insufficiency (yes vs. no)	2.91 (1.19, 7.10)	0.019
Pulmonary hypertension (yes vs. no)	3.98 (1.09–14.5)	0.036
Ventilation		
cPAP vs. room air + venturi mask + low flow	0.90 (0.15–5.36)	0.9
Endotracheal intubation vs. room air + venturi mask + low flow	2.85 (0.74–11.0)	0.13
Empyema/pneumatocele/abscess vs. others	0.17 (0.05–0.58)	0.004
Thoracotomy vs. RATS/VATS	4.90 (1.84–13.1)	0.001

*Age, renal insufficiency, pulmonary hypertension, and thoracotomic approach were found to be independent prognostic risk factors for 30-day mortality. Preoperative endotracheal intubation failed to reach statistical significance, although showing a clear trend toward higher 30-day mortality. Infective pathologies (e.g., empyema/pneumatoceles/lung abscess) compared with others (e.g., pneumothorax, hemothorax, etc.) resulted in a significant protective factor, ensuring lower 30-day mortality.*

### Morbidity

Postoperative complications are reported in Table 6A multivariable adjusted analysis, the only variables statistically significative as predictors for 30-day morbidity were age (adjusted OR 1.05 [95% CI, 1.01, 1.10], *p*-value 0.023) and thoracotomy (adjusted OR 3.85 [95% CI, 1.35, 12.0] *p*-value, 0.014) (Table 6B).

**Table 6A:** Postoperative complications and their frequency.

Complication type	*N* (%)
ARDS	3 (9)
Atrial fibrillation	2 (6)
Bleeding	3 (9)
Celulitis	1 (3)
Fistula	1 (3)
MOF	4 (12)
Prolonged air leaks	7 (21)
Septic shock	7 (21)
Subcutaneous emphysema	5 (15)
Wound infection	1 (3)
Clavien grade of complication (%)	
I	2 (6)
II	7 (21)
IIIa	8 (24)
IVa	3 (9)
V	14 (41)

**Table 6B:** Multivariable logistic regression model on morbidity concerning postoperative complications (*n* = 83, 34 complications).

Variable	OR(95% CI)	*p*-Value
Age (per year)	1.05 (1.01–1.10)	0.023
Gender (males vs. females)	0.91 (0.27–3.14)	0.9
Renal insufficiency (yes vs. no)	3.23 (0.75–16.3)	0.3
Pulmonary hypertension (yes vs. no)	3.60 (0.55–32.2)	0.2
Ventilation		
cPAP vs. room air + venturi + lowflow	2.82 (0.60–14.4)	0.2
Endotracheal intubation vs. room air + venturi + lowflow	1.42 (0.44–4.83)	0.6
Empyema/pneumatocele vs. others	1.56 (0.46–5.31)	0.5
Thoracotomy vs. RATS/VATS	3.85 (1.35–12.0)	0.014

*The only variables found to be statistically significative as predictors for 30-day morbidity were age (adjusted OR 1.05 [95% CI, 1.01, 1.10], p-*v*alue 0.023) and thoracotomy (adjusted OR 3.85 [95% CI, 1.35, 12.0] p-Value, 0.014).*

**Table 7:** Cumulative incidence of in-hospital mortality and discharge by time since surgery estimated by using the Aalen–Johansen estimator with 95% confidence intervals.

Time (days)	Mortality (%)	Discharge (%)
1	2 (0–6)	0 (0–0)
3	4 (0–8)	0 (0–0)
4	5 (0–9)	1 (0–4)
5	10 (3–16)	8 (2–14)
6	10 (3–16)	11 (4–18)
7	11 (4–18)	13 (6–21)
8	13 (6–21)	19 (11–28)
9	16 (8–23)	22 (13–31)
10	16 (8–23)	29 (19–39)
11	17 (9–25)	31 (21–41)
12	18 (10–26)	34 (24–44)
13	18 (10–26)	37 (27–48)
14	19 (11–28)	40 (29–50)
15	20 (12–29)	40 (29–50)
16	20 (12–29)	42 (32–53)
17	23 (14–32)	42 (32–53)
18	24 (15–33)	45 (34–55)
19	24 (15–33)	46 (35–57)
20	25 (16–35)	46 (35–57)
21	27 (17–36)	46 (35–57)
22	27 (17–36)	49 (39–60)
23	27 (17–36)	51 (40–61)
24	27 (17–36)	52 (41–63)
26	27 (17–36)	53 (42–64)
27	28 (18–37)	53 (42–64)
28	28 (18–37)	54 (43–65)
34	28 (18–37)	55 (45–66)
36	29 (19–39)	55 (45–66)
39	30 (20–40)	57 (46–67)
41	31 (21–41)	57 (46–67)
45	31 (21–41)	58 (47–68)
50	33 (22–43)	58 (47–68)

## Discussion

Many recent studies have focused on patients with SARS-CoV-2 infection undergoing elective or emergency surgical procedures. Reports have shown high morbidity and mortality rates (8–12); therefore, it is commonly believed that surgery should be avoided or postponed, whenever possible. The COVID-Surg Collaboration group (9) set up the largest observational study on this topic, considering an international sample of more than 1,000 SARS-CoV-2 positive patients undergoing emergency and elective surgery. They showed 23.8% (268/1,128) mortality rate and 51.25% (577/1,128) pulmonary morbidity rate; 7.6% of surgical procedures (86/1,128) involved the cardiothoracic district. Knisely et al. (8), considering a sample of 36 patients with SARS-CoV-2 infection who underwent urgent and emergent surgical procedures at 2 hospitals in New York City, reported a perioperative morbidity of 58.3% and postoperative mortality of 16.7%. In a recent review by Nahshon et al. (11) who focused on postoperative outcomes of patients diagnosed with COVID-19 during the perioperative period, 27.5% postoperative mortality rate (14/51) and severe pulmonary complications were reported. De Luca et al. (12), whose study involved 68 SARS-CoV-2 positive patients undergoing cancer and emergency surgery in 20 Italian surgical institutions, recorded a postoperative mortality rate of 14.7% (10/68) and a pulmonary complication rate of 33.8% (26/68).

Only a few authors have discussed the burden of COVID-19 in thoracic surgery (13–15) and even fewer have specifically examined thoracic surgical procedures in the management of COVID-19 complications, confining themselves to single case reports (16–20) or limited case series (21, 22); in fact, the general consensus is that surgery in these patients is not advisable. Nevertheless, it is well known that SARS-CoV-2 can lead to dramatic thoracic complications, some of which exclusively need surgical management. Chang et al. (23), describing 13 patients with COVID-19 who underwent surgery for thoracic complications, registered a 30-day mortality rate of 23% and a 30-day morbidity rate of 61%. We assessed the efficacy and safety of surgery in the largest international cohort of patients with COVID-19-related thoracic complications requiring an operative management.

We recorded a 30-day mortality rate of 27.7% and a 30-day morbidity rate of 49%. A rough comparison with outcomes in thoracic surgery before the advent of the pandemic shows dramatic results. Seely et al. (7), based on a dataset of 1,260 thoracic surgical procedures, reported a mortality rate of 2.2% and a morbidity rate of 29.3%, including all types of surgeries performed on all types of patients. These data confirm that surgical management of patients with COVID-19-related thoracic complications is a high-risk procedure and, therefore, should hardly be recommended. However, some other considerations deserve to be drawn.

We compared our results with the literature concerning the same pathologies in patients without SARS-CoV-2 infection, in order to ascertain whether the outcomes similarly worsened for each single affection addressed.

Patients affected by pneumothorax with persistent air leaks secondary to COVID-19 seem to fare worse than those with pneumothorax secondary to interstitial non-infectious lung disease, such as COPD or PFD, for which the in-hospital mortality rate ranges from 1.4% (24) to 5% (25) and from 15% (26) to 21.4% (24), respectively. Nevertheless, these types of patients seem to have a better prognosis than those with pneumothorax secondary to granulomatosis with polyangiitis, where a mortality rate of 40% (27) has been reported.

With regard to the prognosis of patients with pleural empyema, in the last expert consensus statement for surgical management (28), a 30-day mortality rate ranging from 0% to 16.1% was reported. In a recent review, Godfrey et al. (29) suggested that the high variability of these data could be attributed to confused patient selection criteria; for example, the 30-day mortality rate appears to be higher in population-based studies than in single institutions performing up-front VATS decortication in younger patients, less acute and ill patients and patients with fewer comorbidities. Therefore, the prognosis was slightly worse in our series than in patients with unrelated COVID-19 empyema.

Focusing on the 13 patients operated for hemothorax, the registered 30-day mortality rate of 46% seems to be hardly comparable to that reported in the literature concerning patients without COVID-19. In fact, hemothorax is usually a consequence of postoperative or post-traumatic bleeding: the latter usually has a good prognosis with extremely low mortality rates (30) when patients are reoperated early through careful hemostasis, while the former has a wide span of the prognosis depending on the kind of trauma that occurred. Mortality is closely related to traumatic damage and to the vessel involved in bleeding rather than hemothorax itself. In our series, hemothorax was related to iatrogenic factors such as chest tube placement, thoracentesis or previous surgery in 11 patients. The remaining two patients underwent spontaneuos hemothorax or, more likely, it was due to mechanical ventilation, as already described in the literature (31).

The 30-day mortality rate in patients with lung abscess was 40%. In a series of 28 patients with lung abscess undergoing wedge resection, lobectomy or pneumonectomy, Lee and colleagues reported a perioperative mortality rate of 14.3% (32). Huang et al. (33), focusing on a sample of 22 surgical patients, presented a mortality rate of 23%. In both case studies, the mortality rates were clearly lower than those that emerged from our series.

With regard to the causes of death, 13 out of 23 patients died from surgical complications (Clavien V), while 10 probably died from causes apparently not related to surgery but to the underlying COVID-19. Data reveal that this surgery is very demanding but also suggest that prognosis is largely influenced by variables linked to preoperative prognostic factors rather than to the surgery itself.

Notably, age was an independent variable for 30-day mortality, increasing the risk of death within 30 days from surgery by 4%. HR related to advanced age was 1.05. That may apparently seem of irrelevant clinical significance, especially since from the COVIDsurg collaborative study (9) emerged an OR of 2.30 for patients aged 70 years and older. However, while that research included all surgical patients having positivity to SARS-CoV-2 without distinguishing the severity of the disease, we included only patients affected by COVID-19, attenuating the influence of age on the prognosis.

Pulmonary hypertension was found to significantly increase the 30-day mortality risk by more than four times in our population. This variable has not been investigated in previous studies, but we think it could be of primary importance, based on the pathophysiology of COVID-19. Indeed, the latest research has focused attention on the production of self-antibodies against Annexin-A2 as a leading mechanism on the basis of the severe form of this disease (34, 35), which should be explained by the critical role of this phospholipid-binding protein in lung elasticity, fibrinolysis and integrity of the pulmonary vascular system (36), converging toward an increase in pulmonary artery pressure. In our series, pulmonary hypertension, when recorded, was an anamnestic event already present before COVID-19 onset. All cases were studied with cardiac ultrasound, but unfortunately, we did not have sufficient data for making a comparison with the prehospitalization situation. We strongly suggest assessing the pulmonary artery pressure, possibly through non-invasive echocardiography, before surgery.

Renal failure, showing a trend toward an increasing risk of death of almost three times, was confirmed as a critical prognostic factor. This is not surprising, as acute kidney failure is known to be associated with high morbidity and mortality.

Endotracheal intubation failed to achieve statistical significance; however, in our opinion, it is a prognostic factor worthy of consideration. Indeed, patients intubated before surgery had a postoperative risk of death of almost three times. This finding could be related to the fact that patients deserving intubation before surgery were already in an advanced stage of disease at the peak of cytokines storm, thus conditioning surgical outcomes. The lack of statistical significance could be explained by the low sample size and number of events. Therefore, we suggest being extremely careful in operating these patients, adopting whenever possible alternative therapies that could decrease the inflammatory response before proceeding to surgery.

On the other hand, in our multivariable analysis, patients who underwent surgery for infective affections (empyema/infected pneumatoceles/lung abscess) had a significant lower risk of death, with a reduction in mortality risk of about 83%, suggesting that it can be considered an independent protective factor. This seems quite surprising and could be justified by the minimal invasive approach most often used in the first group. However, no homogeneous preoperative general conditions in the two groups could represent a bias.

We also studied the risk factors for 30-day morbidity. On multivariate logistic analysis, age was confirmed to be also an independent prognostic factor for postoperative morbidity, increasing the risk of complications to 5%. We explain the slight difference behind the mortality rate, but the statistical significance of this variable strengthens the validity of our study. Interestingly, thoracotomy was found to be an independent 30-day prognostic factor for mortality and morbidity as well, with 5-fold and 4-fold increases of the risk of death and complications, respectively, compared with minimally invasive techniques such as VATS or RATS. The advantages of minimally invasive procedures over the classic ones performed through thoracotomy are now well established in thoracic surgery (37), but we think that our result is biased by patient selection, since VATS and RATS were reserved only for less critically ill patients, and, hence, they were less prone to develop postoperative complications and deaths.

### Limitations

Our series has several limitations. First of all, given the retrospective nature of this study, intrinsic patient selection bias could have occurred. Secondly, the sample size, although consistent, remained small, with a limited follow-up period, which only allowed us to investigate early outcomes. Unlikely, we could not determine the total number of COVID-19 patients hospitalized in each participating center, so we were unable to provide the percentage of patients requiring surgery over the total number. Moreover, we could not find adequate controls to be compared with our patients, such as those with COVID-19-related complications not surgically managed, thus weakening our results and constraining us from drawing conclusions over which is the best treatment. Finally, while the choice of conducting a multicenter international study allowed us to generalize our findings, such an exercise could be biased by the absence of standardized indications for surgery and for technical procedures. In particular, the lack of homogeneous data made it impossible to determine how “serious” was COVID-19 infection at the time of surgery.

Furthermore, our study covers a period between the beginning of the vaccination campaign and the ongoing exercise, enrolling both vaccinated and unvaccinated patients. This may be a confounding factor on outcomes.

Another bias concerns the lack of information about the use of vv-ECMO before or during surgery; this a hot topic, as it is commonly suggested that COVID-19 patients who develop complications like empyema or hematothorax on vvECMO-support are particularly challenging to manage. The role of ECMO support in the treatment of severe COVID-19 has been addressed by many authors in the last 2 years, concluding that it should be considered as a rescue therapy for patients with refractory hypoxemia, despite lung-protective ventilation. The criteria for optimizing outcomes have been well described by Hong and co-workers (38). Unfortunately, we have not been able to collect data on ECMO in our series of cases, but one patient underwent surgery on ECMO support at our hospital. This patient underwent surgery for pleural empyema, and intraoperative ECMO support was given because of severe respiratory distress on mono-pulmonary ventilation.

However, to our knowledge, this is the largest study on thoracic surgery in COVID-19 patients to date.

## Conclusion

Prognosis for COVID-19-related thoracic complications requiring surgical management is poor and surgery is affected by much higher mortality and morbidity rates than those conventionally accepted in patients without COVID-19. Nevertheless, the reported 30-day survival rate seems to be still satisfactory for ruling out preventive surgery, mainly because mortality is largely influenced by events that are linked to baseline pathological conditions rather than to the surgery itself. Surgery should be considered only in selected patients, with a salvage intent. In particular, prognosis seems to be better for infectious complications such as pleural empyema or infected pneumatoceles than for bleeding complications such as hemothorax or hemoptysis. Moreover, younger individuals without pulmonary hypertension or renal failure appear to have a better prognosis. We suggest a careful management of patients needing preoperative intubation. We strongly recommend a multidisciplinary approach to produce tailored solutions.

## Data Availability

This article can be distributed in accordance with the Creative Commons Attribution No Commercial (CC BY-NC 4.0) license, which permits others to distribute, remix, adapt, build upon this work non-commercially, and license their derivative works on different terms, provided the original work is properly cited and the use is non-commercial See: http://creativecommons.org/licenses/by-nc/4.0/.
